# Supplementary material for: Superspreading of airborne pathogens in a heterogeneous world
Source: Sci Rep. 2021 May 27;11:11191. doi: 10.1038/s41598-021-90666-w (PMC8160272; doi:10.1038/s41598-021-90666-w)
Supplement: Supplementary file 1 — Supplementary Information. [file 41598_2021_90666_MOESM1_ESM.pdf]

SI: Superspreading of Airborne Pathogens in a Heterogeneous World

Julius B. Kirkegaard (julius.kirkegaard@nbi.ku.dk), Joachim Mathiesen, and Kim Sneppen
Niels Bohr Institute, University of Copenhagen, 2100 Copenhagen, Denmark
(Dated: March 23, 2021)

Distribution

Marginalizing over

$$P_N(n) = (\nu - 1)\frac{a^{\nu-1}}{(a+n)^\nu} \tag{1}$$

$$P_{Z_i}(z) = \frac{(\alpha n)^z e^{-\alpha n}}{z!} \tag{2}$$

gives

$$P_Z(z) = \frac{(\nu-1)a^{\nu-1}}{z!}\int_0^\infty \frac{(\alpha n)^z e^{-\alpha n}}{(a+n)^\nu}\,\mathrm{d}n \equiv \frac{(\nu-1)a^{\nu-1}}{z!} f(z,\nu). \tag{3}$$

We only need the integral for integer values of z, so we may do the calculation recursively:

$$\begin{aligned} f(z+1,\nu) &= \int_0^\infty \frac{(\alpha n)^{z+1} e^{-\alpha n}}{(a+n)^\nu} \\ &= \alpha \int_0^\infty \frac{n(\alpha n)^z e^{-\alpha n}}{(a+n)^\nu} \\ &= \alpha\left[\int_0^\infty \frac{(a+n)(\alpha n)^z e^{-\alpha n}}{(a+n)^\nu} - a\int_0^\infty \frac{(\alpha n)^z e^{-\alpha n}}{(a+n)^\nu}\right] \\ &= \alpha\left[\int_0^\infty \frac{(\alpha n)^z e^{-\alpha n}}{(a+n)^{\nu-1}} - a\int_0^\infty \frac{(\alpha n)^z e^{-\alpha n}}{(a+n)^\nu}\right] \\ &= \alpha\left[f(z,\nu-1) - a f(z,\nu)\right]. \end{aligned} \tag{4}$$

With this recursion, all values $0 \le z \le n$ can be calculated in $\mathcal{O}(n^2)$ evaluations if we know $f(0,\nu)$. Luckily this integral is much simpler:

$$f(0,\nu) = e^{a\alpha}\,\alpha^{\nu-1}\,\Gamma(1-\nu, a\alpha) = e^{a\alpha}\,a^{1-\nu}\,E_\nu(a\alpha), \tag{5}$$

where $\Gamma(\cdot,\cdot)$ is the incomplete gamma function and $E_n(\cdot)$ is the exponential integral function. Finally we solve the recursion to obtain the result of the main text.

Exponential Distribution

When the method of disease transmission is by physical touch, the exponential distribution becomes more relevant. Taking

$$P_N(n) = s^{-1}e^{-n/s} \tag{6}$$

we find

$$P_{Z_i}(z) = (\alpha s)^z\,(1+\alpha s)^{-(z+1)}. \tag{7}$$

In this case we can directly solve for the distribution for any M. Calculating the probability generating function, we find

$$G_{Z_i}(\zeta) = \frac{1}{1+\alpha s(1-\zeta)}. \tag{8}$$

We raise this to the M-th power and take z derivatives to find

$$P_Z(z) = \binom{M+z-1}{z} \frac{(\alpha s)^z}{(1+\alpha s)^{M+z}}. \tag{9}$$

This has average $\alpha M s$, which for fixed R_0 implies $\alpha = R_0/(sM)$. Thus the distribution is independent of s for fixed R_0:

$$P_Z(z) = \binom{M+z-1}{z} \frac{(R_0/M)^z}{(1+R_0/M)^{M+z}}. \tag{10}$$

This is a negative binomial distribution, $\mathrm{NB}(M,(1+R_0/M)^{-1})$. It corresponds to the superspreading distribution of Ref. [1] if one identifies their dispersion parameter k with M. The correspondence arises because the sum of M exponentially distributed random numbers is gamma distributed and the sum of Poisson distributed random numbers is itself Poisson distributed.

Since the distribution is independent of s, so be its output statistics. In particular, the above distribution gives $f_{20} \approx 0.60$ for $M = 1$ and $R_0 = 2.0$, whereas we have $f_{20} \approx 0.65$ for $R_0 = 1.0$. For $M = 5$ the fractions fall to $f_{20} \approx 0.46$ ($R_0 = 2.0$) and $f_{20} \approx 0.54$ ($R_0 = 1.0$). Since these are valid for all s, we conclude that extreme statistics such as 20 % infecting 80 % can never be achieved from exponential distributions.

Mitigation

For mitigation we redistribute people below a maximum $n = x$. So we have

$$P_N(n) = \frac{1}{\int_0^x \frac{1}{(a+n)^\nu}\,\mathrm{d}n} \frac{1}{(a+n)^\nu} \equiv \frac{1}{N(a,\nu,x)} \frac{1}{(a+n)^\nu} \tag{11}$$

where the normalization is a simple, but ugly integral. We now need

$$P_Z(z) = \frac{1}{z!N(a,\nu,x)} \int_0^x \frac{(\alpha n)^z e^{-\alpha n}}{(a+n)^\nu}\,\mathrm{d}n \equiv \frac{1}{z!N(a,\nu,x)} f_x(z,\nu). \tag{12}$$

By the exact same argument as above, we have

$$f_x(z+1,\nu) = \alpha\left[f_x(z,\nu-1) - a f_x(z,\nu)\right]. \tag{13}$$

Again we need the value for $z = 0$, which in this case is

$$f_x(0,\nu) = e^{a\alpha}\,\alpha^{\nu-1}\left(\Gamma(1-\nu,a\alpha) - \Gamma(1-\nu,(a+x)\alpha)\right). \tag{14}$$

.

Exponential Cutoff

We use a power law for the distribution of sizes of location. This creates two problems: (1) the model becomes invalid as $\nu \to 2$ and (2) higher order moments diverge. To fix these issues an exponential cutoff must be included. In this case we consider

$$P_N(n) = \frac{a^{\nu-1}}{E_\nu(a/s)} \frac{e^{-\frac{a+n}{s}}}{(a+n)^\nu}, \tag{15}$$

where s denotes the (soft) cutoff value. Now we have

$$P_Z(z) = \frac{a^{\nu-1}}{z!E_\nu(a/s)} \int_0^\infty \frac{(\alpha n)^z e^{-\alpha n} e^{-\frac{a+n}{s}}}{(a+n)^\nu}\,\mathrm{d}n \equiv \frac{a^{\nu-1}}{z!E_\nu(a/s)} f_s(z,\nu), \tag{16}$$

and again

$$f_s(z+1,\nu) = \alpha\left[f_s(z,\nu-1) - a f_s(z,\nu)\right], \tag{17}$$

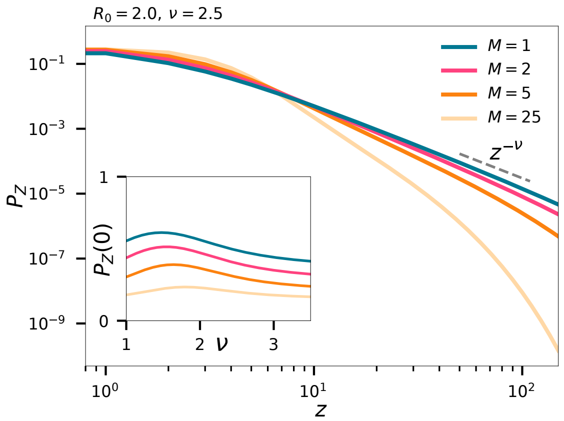

FIG. 1. Same as main text, but with exponential cutoff $s = 1{,}000$.

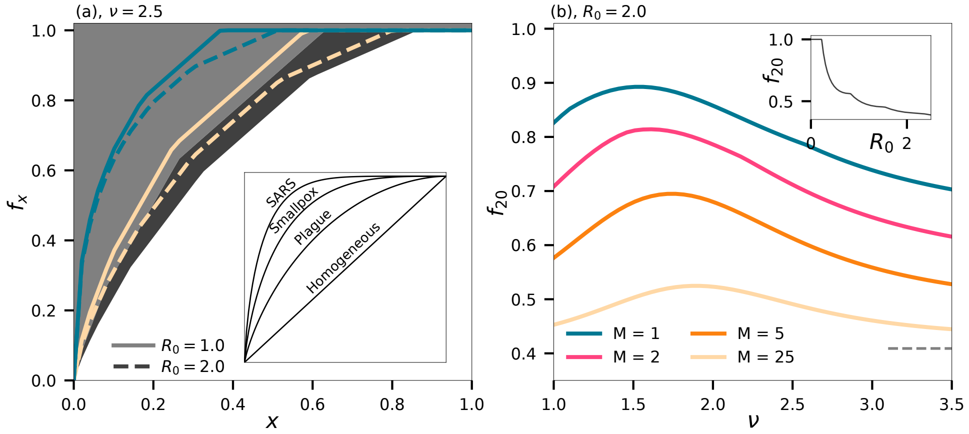

FIG. 2. Same as main text, but with exponential cutoff $s = 1{,}000$.

where now

$$f_s(0,\nu) = e^{a\alpha}\, a^{1-\nu}\, E_\nu(a\alpha + as^{-1}). \tag{18}$$

Solving this recursion leads to

$$P_Z(z) = \frac{(-a\alpha)^z e^{a\alpha}}{z!\, E_\nu(a/s)} \sum_{j=0}^{z} (-1)^j \binom{z}{j} E_{\nu-j}(a\alpha + as^{-1}). \tag{19}$$

For $\nu \geq 1$, we have $1/E_\nu(0) = (\nu - 1)$, confirming that this result reduces to that of the main text for $s \to \infty$. Note that for s finite, we no longer need to require $\nu > 2.0$. Fig. 3 shows the plot f_{20} with an exponential cutoff. We note that the values of $\nu = 2.5$ are virtually unchanged. Interestingly, the curves now yield a maximum. Thus, with a cutoff, the superspreading statistics emerging from spatial heterogeneity can be bounded even if ν is unknown.

Mitigation & Exponential Cutoff

We now have

$$P_N(n) = \frac{1}{\int_0^x \frac{e^{-n/s}}{(a+n)^\nu}\,\mathrm{d}n}\,\frac{e^{-n/s}}{(a+n)^\nu} \equiv \frac{1}{N(a,\nu,x)}\,\frac{e^{-n/s}}{(a+n)^\nu}. \tag{20}$$

As before this leads us to

$$P_Z(z) = \frac{1}{z!N(a,\nu,x)} \int_0^x \frac{(\alpha n)^z e^{-n/s} e^{-\alpha n}}{(a+n)^\nu}\,\mathrm{d}n \equiv \frac{1}{z!N(a,\nu,x)} f_x(z,\nu). \tag{21}$$

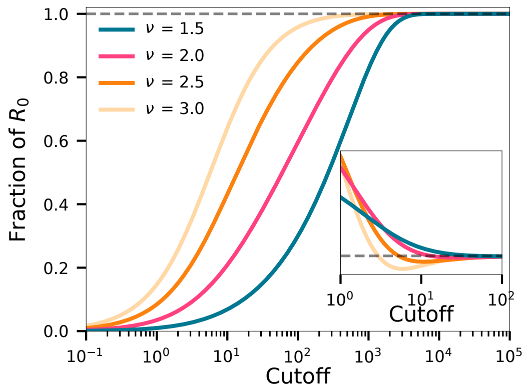

FIG. 3. Same as main text, but with exponential cutoff $s = 1{,}000$.

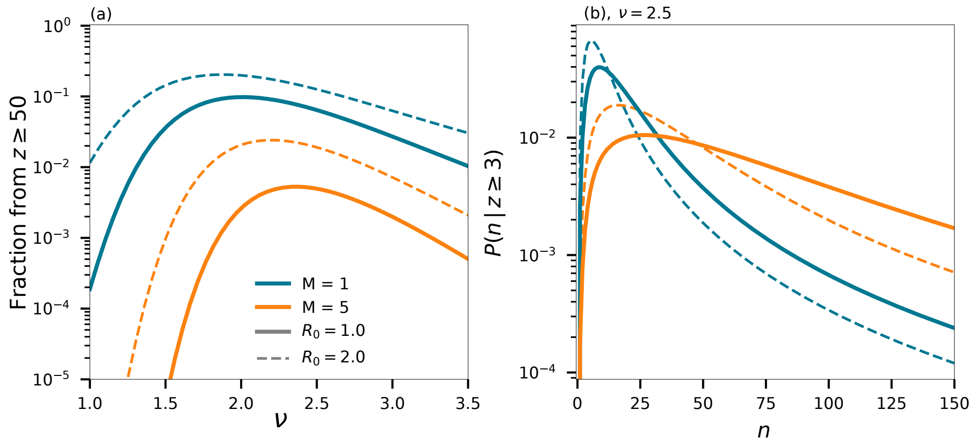

FIG. 4. Same as main text, but with exponential cutoff $s = 1{,}000$.

By the exact same argument as above, we have

$$f_x(z+1,\nu) = \alpha\left[f_x(z,\nu-1) - af_x(z,\nu)\right]. \tag{22}$$

Again we need the value for $z = 0$, which in this case is

$$f_x(0,\nu) = \frac{s\,(\alpha+s^{-1})^{\nu}e^{a\alpha+as^{-1}}}{1+\alpha s}\left[\Gamma(1-\nu, a\alpha+as^{-1}) - \Gamma(1-\nu,(a+x)(1+s\alpha)s^{-1})\right]. \tag{23}$$

.

[1] J. O. Lloyd-Smith, S. J. Schreiber, P. E. Kopp and W. M. Getz. Superspreading and the effect of individual variation on disease emergence. *Nature*, 438, 355–35, 2005.
